# Supplementary material for: Clinical Progression on CDR-SB©: Progression Free Time at Each 0.5-unit Level in Dominantly Inherited and Sporadic Alzheimer’s Disease Populations
Source: medRxiv. 2025 Feb 20:2025.02.17.25322322. Preprint. [Version 1] doi: 10.1101/2025.02.17.25322322 (PMC11875246; doi:10.1101/2025.02.17.25322322)
Supplement: Supplement 1 [file NIHPP2025.02.17.25322322v1-supplement-1.pdf]

medRxiv preprint doi: https://doi.org/10.1101/2025.02.17.25322322; this version posted February 20, 2025. The copyright holder for this preprint (which was not certified by peer review) is the author/funder, who has granted medRxiv a license to display the preprint in perpetuity.
It is made available under a CC-BY 4.0 International license .

Supplemental Materials

Parametric disease progression model (DPM)

Briefly, the model applied to the DIAN study can be described as:

$$Y_{ij} = (\beta + \mu_i) + \gamma_1 e^{\left(\frac{EYO_{ij}+\delta_i}{\gamma_2}\right)} + \varepsilon_{ij}$$

Where,

- y_{ij} denotes the assessment for subject i at visit j (EYO_{ij})
- β is the mean of the healthy period (e.g. EYO <=-15)
- γ_1 is the shape parameter and influences how much steep the curve exhibits. It also represents the mean decline from the healthy period to symptom onset (e.g. EYO=0)
- γ_2 represents the scale parameter, and affects the rate at which the exponential function grows or decays
- μ_i is the subject-level random effect that shifts the individual data up or down on the y-axis, accounting for individual variability in the baseline or intercept of the response variable
- δ_i is the subject-level random effect that shifts the individual data left or right on the x-axis, adjusting for individual variability in the timing or progression from symptom onset
- μ_i and δ_i are assumed to follow a bivariate normal distribution: $\begin{pmatrix} u_i \\ \delta_i \end{pmatrix} \sim N\left(\mathbf{0}, \begin{bmatrix} \sigma_{u_i}^2 & \sigma_{u_i\delta_i} \\ \sigma_{u_i\delta_i} & \sigma_{\delta_i}^2 \end{bmatrix}\right)$
- ε_{ij} is the within-subject error and is assumed to follow a normal distribution $N(0, \sigma_0^2)$ for participants with CDR 0 at baseline and $N(0, \sigma_1^2)$ for participants with CDR>0.

For ADNI study, the same model is applied with EYO being replaced by the time since enrollment in years.

After the DPM is estimated, the progression time to each CDR-SB level was calculated based on the model. Then the progression free time was calculated as the difference between the progression time corresponding to two consecutive CDR-SB levels.

Supplemental Table 1: Estimated progression-free time for each 0.5-unit CDR-SB interval for each study

	**DIAN Study**				**ADNI Study**			
**CDR-SB Interval**	**Estimate**	**SE**	**Lower**	**Upper**	**Estimate**	**SE**	**Lower**	**Upper**
**0.5 to 1**	1.647	0.041	1.567	1.728	2.250	0.071	2.106	2.387
**1 to 1.5**	0.960	0.021	0.919	1.001	1.350	0.044	1.268	1.441
**1.5 to 2**	0.680	0.014	0.652	0.708	0.970	0.033	0.908	1.037
**2 to 2.5**	0.527	0.011	0.506	0.549	0.760	0.026	0.708	0.811
**2.5 to 3**	0.431	0.009	0.413	0.448	0.620	0.022	0.580	0.666
**3 to 3.5**	0.364	0.008	0.349	0.379	0.530	0.019	0.492	0.565
**3.5 to 4**	0.315	0.007	0.302	0.328	0.460	0.016	0.426	0.491
**4 to 4.5**	0.278	0.006	0.267	0.289	0.410	0.015	0.376	0.434
**4.5 to 5**	0.249	0.005	0.238	0.259	0.360	0.013	0.337	0.389
**5 to 5.5**	0.225	0.005	0.216	0.234	0.330	0.012	0.305	0.352

medRxiv preprint doi: https://doi.org/10.1101/2025.02.17.25322322; this version posted February 20, 2025. The copyright holder for this preprint (which was not certified by peer review) is the author/funder, who has granted medRxiv a license to display the preprint in perpetuity.
It is made available under a CC-BY 4.0 International license .

**5.5 to 6**	0.205	0.004	0.197	0.214	0.300	0.011	0.279	0.322
**6 to 6.5**	0.189	0.004	0.181	0.197	0.280	0.010	0.256	0.297
**6.5 to 7**	0.175	0.004	0.168	0.182	0.260	0.009	0.238	0.275
**7 to 7.5**	0.163	0.003	0.156	0.169	0.240	0.009	0.221	0.256
**7.5 to 8**	0.152	0.003	0.146	0.158	0.220	0.008	0.207	0.240
**8 to 8.5**	0.143	0.003	0.137	0.149	0.210	0.008	0.194	0.225
**8.5 to 9**	0.135	0.003	0.129	0.140	0.200	0.007	0.183	0.212
**9 to 9.5**	0.128	0.003	0.122	0.133	0.190	0.007	0.174	0.201
**9.5 to 10**	0.121	0.003	0.116	0.126	0.180	0.007	0.165	0.191
**10 to 10.5**	0.115	0.002	0.110	0.120	0.170	0.006	0.157	0.182
**10.5 to 11**	0.110	0.002	0.105	0.114	0.160	0.006	0.149	0.173
**11 to 11.5**	0.105	0.002	0.100	0.109	0.150	0.006	0.143	0.166
**11.5 to 12**	0.100	0.002	0.096	0.105	0.150	0.006	0.137	0.159
**12 to 12.5**	0.096	0.002	0.092	0.100	0.140	0.005	0.131	0.152
**12.5 to 13**	0.092	0.002	0.089	0.096	0.140	0.005	0.126	0.146
**13 to 13.5**	0.089	0.002	0.085	0.093	0.130	0.005	0.121	0.141
**13.5 to 14**	0.086	0.002	0.082	0.089	0.130	0.005	0.117	0.136
**14 to 14.5**	0.083	0.002	0.079	0.086	0.120	0.005	0.113	0.131
**14.5 to 15**	0.080	0.002	0.077	0.083	0.120	0.004	0.109	0.126
**15 to 15.5**	0.077	0.002	0.074	0.080	0.110	0.004	0.105	0.122
**15.5 to 16**	0.075	0.002	0.072	0.078	0.110	0.004	0.102	0.118
**16 to 16.5**	0.073	0.002	0.070	0.076	0.110	0.004	0.099	0.115
**16.5 to 17**	0.070	0.001	0.067	0.073	0.100	0.004	0.096	0.111
**17 to 17.5**	0.068	0.001	0.065	0.071	0.100	0.004	0.093	0.108
**17.5 to 18**	0.066	0.001	0.064	0.069	0.100	0.004	0.091	0.105
